# Mechanisms of Immunosuppressive Tumor Evasion: Focus on Acute Lymphoblastic Leukemia

**DOI:** 10.3389/fimmu.2021.737340

**Published:** 2021-11-18

**Authors:** Silvia Jiménez-Morales, Ivan Sammir Aranda-Uribe, Carlos Jhovani Pérez-Amado, Julian Ramírez-Bello, Alfredo Hidalgo-Miranda

**Affiliations:** ^1^ Laboratorio de Genómica del Cáncer, Instituto Nacional de Medicina Genómica, Mexico City, Mexico; ^2^ Departamento de Farmacología, División de Ciencias de la Salud, Universidad de Quintana Roo, Quintana Roo, Mexico; ^3^ Programa de Doctorado en Ciencias Bioquímicas, Universidad Nacional Autónoma de México, Mexico City, Mexico; ^4^ Departamento de Endocrinología, Instituto Nacional de Cardiología Ignacio Chávez, Mexico City, Mexico

**Keywords:** acute lymphoblastic leukemia, immunoediting, immunotherapy, tumor immune evasion, immune cells

## Abstract

Acute lymphoblastic leukemia (ALL) is a malignancy with high heterogeneity in its biological features and treatments. Although the overall survival (OS) of patients with ALL has recently improved considerably, owing to the application of conventional chemo-therapeutic agents, approximately 20% of the pediatric cases and 40–50% of the adult patients relapse during and after the treatment period. The potential mechanisms that cause relapse involve clonal evolution, innate and acquired chemoresistance, and the ability of ALL cells to escape the immune-suppressive tumor response. Currently, immunotherapy in combination with conventional treatment is used to enhance the immune response against tumor cells, thereby significantly improving the OS in patients with ALL. Therefore, understanding the mechanisms of immune evasion by leukemia cells could be useful for developing novel therapeutic strategies.

## Introduction

Acute lymphoblastic leukemia (ALL) is a group of lymphoid neoplasms derived from B- and T-lymphoid progenitors that are clinically and genetically heterogeneous (1–5). The incidence of ALL is rapidly growing worldwide, and it is estimated to be one in 100,000 persons/year globally (6, 7), with a peak prevalence between 1 and 4 years old (7, 8) and during the fifth decade of life (5, 9, 10). The overall survival (OS) for pediatric patients is >90% in high-income countries but is lower in middle- and low-income countries (11)—for instance, in Mexico, the global survival rate reported of children with ALL was 63.9%, the event-free survival rate was 52.3% after an average follow-up of 3.9 years (12), and it had a high rate of early mortality (12.1%) (13). Unfortunately, OS in adults with ALL is worst. Even though most adult patients can reach initial complete remission using recently developed treatments, only 40–50% (<20% in patients aged 60 years or older) of the 5-year OS is achieved (5, 14–17). Relapse, defined as the return of the disease in patients who reach initial complete remission, is one of the main obstacles in achieving improved survival rates (18) and occurs in approximately 20% of children and >50% of adults (19, 20). Most relapse incidences appear during treatment (early relapse: <30 months after diagnosis) or after treatment completion (late relapse: <2 years). Despite the use of diverse anticancer agent combinations (chemotherapy, radiotherapy, and allogeneic hematopoietic stem cell transplantation), patients who experience relapse have a higher probability of treatment failure and death (21). The survival rate of relapsed patients is approximately 50% and worse in relapsed cases where the central nervous system is affected (22–24). Cancer treatment has been based on the use of chemotherapeutic agents that are unable to differentiate between normal and cancer cells. Emerging therapeutic schemes to treat leukemia are based on the knowledge that the immune system plays an important role in tumor cell identification and elimination (25–28). In order to develop new anti-leukemic therapies, it is necessary to understand the mechanisms underlying the displacement of transformed hematopoietic cells by normal hematopoietic progenitors and the immune evasion processes by which the tumor cells hijack the immune system (25, 26). This review focuses on the mechanisms of immune system evasion of ALL cells and its potential for developing new treatments.

## Immune System and Tumor Evasion

The human immune system comprises leukocytes, bone marrow (BM), and other organs. Leukocytes include neutrophils, monocytes, eosinophils, basophils, dendritic cells, lymphocytes (T and B cells), and natural killer (NK) cells. By discriminating self from non-self, the human immune system is responsible for protecting the body from diseases caused by exogenous and endogenous agents. To differentiate between self or non-self, the immune system employs fundamental biochemical differences among cells, such as the absence of methylated cytosine residues in DNA and glycoprotein composition (29, 30). The two immune responses recognized are innate and acquired/adaptive (cell-mediated immunity and humoral immunity). The innate immune response, which is present from birth, activates a non-specific immune response in the presence of self-molecules, such as endogenous damage-associated molecular patterns (DAMPs), Toll-like receptor ligands, and non-self-molecules in a cytokine release-dependent manner (31). Acquired immune response involves antibody production by B cells and antigen presentation to T helper cells to stimulate cytotoxic T cells (CTLs), also known as CD8 + T cells, which induce the elimination of non-self elements and produce immune memory cells (30).

**Figure 1:**
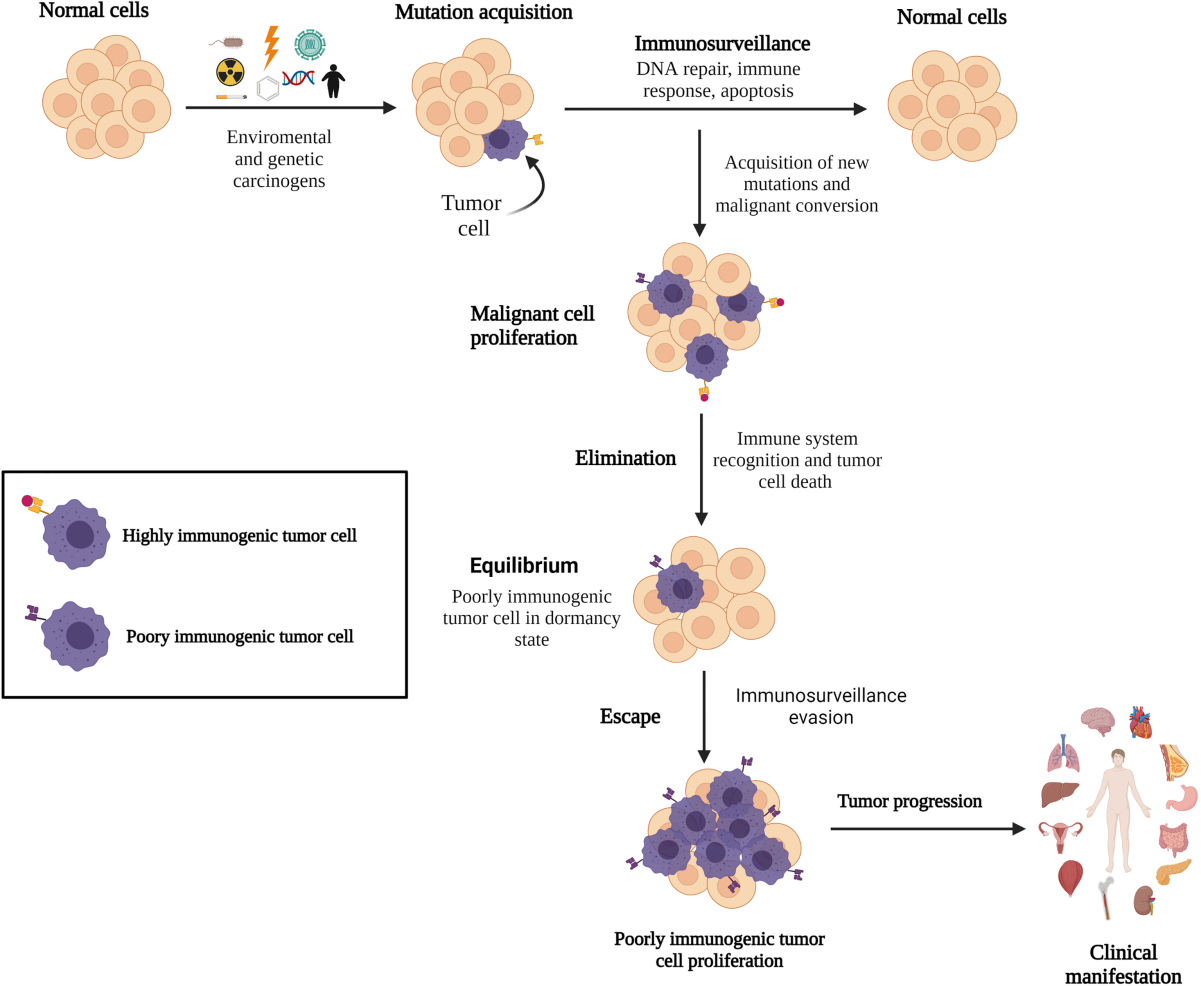
Immune surveillance and cancer development. Emerging malignant cells are identified and eliminated by the immune system; however, certain acquired gene mutations in tumor cells allow them to remain undetected by the immune surveillance system, resulting in cancer.

The cell-mediated immunity is activated when a specific CTL is stimulated to initiate the lysis of pathogens, infected cells, and tumor cells; thus, this protects the body against infection and tumor growth, spreading, and metastasis (31). To prevent tumor emergence, the immune system eliminates oncogenic viral infections, induces the inflammatory microenvironment, and destroys malignant cells (32, 33). Although tumor cells are self in origin, they differ from their normal counterparts in their biochemical and antigenic characteristics and biological behavior. Cancer cells express tumor-specific neoantigens that arise from an inefficient DNA damage repair system and which are presented to the CTLs by the human leukocyte antigen (HLA) system class I. Then, tumor cells are killed through a combination of direct perforin-dependent destruction and by increasing tumor immune sensitivity through the release of inflammatory cytokines, such as interferon (IFN) alpha (INF-α) and tumor necrosis factor (TNF) (30, 34, 35). However, tumor cells have an acquired mechanism to evade the immune system to avoid their destruction (**Figure 1**).

In 1908, Ehrlich proposed the role of the immune system in controlling cancer development. Later, Burnet (1957) suggested that lymphocytes are tasked with identifying and eliminating mutated cells (29, 30) and the presence of an immunological mechanism for eliminating or inactivating potentially dangerous mutant cells before tumor clinical manifestations, a concept called “immunological surveillance” (36). Currently, the role of the immune system in malignant cell elimination is unequivocally established. The quality control of the immune system to fight against tumor cells involves immune cells and their associated molecules, with the CTLs and NK cells being the major components (34, 37). These cells act as tumor suppressors by patrolling the human body and destroying transformed cells before tumor progression. The anti-tumor immune response attacks the tumor through activated lymphocytes to trigger apoptosis by producing perforin and granzyme B to damage the extracellular membrane and enter targeted tumor cells, expressing Fas-L, TNF-related apoptosis-inducing ligand, and IFN-gamma (IFN-γ) (38–40). However, tumor cells can evade immune surveillance mechanisms, and indirectly, the immune system selects tumor cells that carry mutations in genes involved in the immune detection and elimination pathways, leading to cancer progression (**Figure 1**). Despite that CTLs detect tumor cells, they frequently fail to control tumor growth (41, 42). CTL dysfunction could be induced by a continuous stimulation of the tumor antigens and by an immunosuppressive tumor microenvironment (TME), driving the T cells to a functionally exhausted state and cancer progression (42, 43). The interaction between the immune system and cancer establishment is called immunoediting.

The central assumption of immunoediting is that CTLs recognize tumor antigens and drive immunological tumor elimination or model cancer development before re-emerging. This process could select cancer cells with mutations that confer resistance to immune effectors and survival advantages in a tumorigenic environment (44, 45). Immunoediting comprises three phases: elimination, equilibrium, and evasion (30). These mechanisms have been extensively reviewed elsewhere; thus, they are briefly summarized here.

*Elimination* phase involves the recognition and killing of transformed cells and nascent tumors by the immune system through antibody production. This process starts with the recruitment of macrophages, dendritic cells, and infiltrating lymphocytes (NK and natural killer T cells) into the tumor site (46) to suppress angiogenesis and induce immunogenic necrotic tumor cell death, promote regulatory T cells (Tregs) apoptosis, and induce M1 pro-inflammatory macrophage activity to defeat tumor progression (47). Moreover, INF-γ and interleukin-12 (IL-12) enhance cytotoxic responses by NK and CTL cells, promoting tumor death by apoptosis and the release of reactive oxygen and nitrogen intermediates (47). Tumor-specific CD8+ and CD4+ T cells infiltrate the tumor site after the recognition of tumor-specific or tumor-associated antigens through HLA class I and class II molecules, respectively, which facilitate the immune mechanisms in synergy with B cells. Cancer cells that are not eradicated during the elimination phase remain in dormancy or equilibrium (**Figure 1**) (33, 43).

*Equilibrium* is the longest phase where cancer remains clinically undetectable, suggesting that tumor cells coexist with the immune system for up to several years (47, 48). Evidences have shown that immune-mediated cancer dormancy is regulated by CD8+ and CD4+ T cells and IFN-γ (49, 50). Through IFN-γ/STAT1 pathway activation, IFN-γ inhibits tumor cell proliferation and establishes tumor dormancy without destroying malignant cells (30). However, IFN-γ can facilitate tumor escape and relapse by inducing tumor antigen loss, upregulating programmed death 1 (PD1) ligand (PD-L1) in tumor cells and recruiting myeloid-derived suppressor cells (MDSCs) and tumor-associated macrophages (TAMs) to the tumor site (50).

*Escape* is the phase where tumor cells that have evaded the immune surveillance system acquired additional DNA mutations and epigenetic changes and have great effectiveness to proliferate and evade apoptotic mechanisms (51). Although new mutations could drive the expression of tumor-specific antigens that are recognized by CTL cells (52), tumor cell-intrinsic alterations and TME modifications (*e*.*g*., nutrient depletion, metabolic stress, and cytokine regulation) lead to poor immune response and tumor progression (53). Long-term glucose deficiency in the TME results in low T cell response, cytokine production impairment, T cell “anergy” state, and T cell autophagy to save energy (54, 55). Lipid reduction may result in a lower tryptophan concentration in the extracellular environment, which can inhibit CTL proliferation (47, 56). Acquisition of gain-of-function mutations by tumor cells could lead to low or lack of antigenicity properties, resulting in hijacking of immune mechanisms. Mutations can also induce abnormal HLA expression or antigen processing machinery dysregulation; in fact, HLA class I downregulation is described in 40–90% of human tumors (45, 51). Altered PD-1 or PD-L1 expression on tumor and host cells is also observed, which can inhibit T cell activation and enhance the immune tolerance of malignant cells, facilitating tumor immune escape (57, 58). Chronic PD-L1 expression, predominantly by TAMs, prolongs the immunosuppressive TME, likely by tumor-specific T cells, as if they were malignant cells (57). Studies in ALL have shown that leukemic blasts express ligands for NK cell receptors, the natural killer group 2 member D (NKG2D) and DNAX accessory molecule-1, to avoid their destruction (59). Low numbers and impaired NK-cell-mediated cytotoxicity could be due to a reduced level of activating receptors (NKp46, NKp30, NKp44, and NKG2D). Cancer cells can also alter NK cell function by modulating the NK cell surface receptors, releasing soluble factors with immunosuppressive properties such as IL-10 and transforming growth factor beta (TGF-β). The signaling lymphocytic activation molecule-associated protein adaptor, in addition to the overexpression of human leukocyte antigen G (HLA-G), which induced immune tolerance and decreased NKG2D expression in NK cells, contributes to the escape of leukemia cells from immune surveillance (60, 61). Thus, the immune system indirectly promotes tumor progression through the selection of poorly immunogenic malignant clones (44, 45). **Table 1** lists the general mechanisms involved in the evasion phase.

**Table 1:** Potential mechanisms of tumor immune evasion.

Mechanism	Features	Tumor types	References
Malignant cell selection	Low effectiveness to eliminate mutated cells	ALL, breast, bladder, colorectal, CML, esophageal, endometrial, HN, hepatocellular, gastric, glioblastoma, lung, lymphoma, melanoma, pancreatic, prostate, ovarian	(62–67)
	Gain of DNA and epigenetic mutations that increase the proliferation ability		
	Resistance to immunity-induced apoptosis (by abnormal function of IFNγ receptor or tyrosine kinases association)		
Altered expression of HLA antigens and co-stimulatory molecules	Reduced of HLA-I antigen expression	ALL, CLL, AML, CML, breast, cervical, colorectal, gastric, hepatocellular, lymphoma, lung, melanoma	(55, 60, 68, 69)
	Abnormal expression of co-stimulatory molecules (CD80 or CD86)		
	Poor stimulation of T cells		
	Reduced CTL response		
Chronic PD-L1 expression by host cells	Prolongated immunosuppressive state in the tumor microenvironment	ALL, CML, breast, colorectal, esophageal, gastric, HN, lung, melanoma, ovarian, sarcoma	(55, 57, 58, 70, 71)
	Repressed T-cell function		
T cell dysfunction	Reduced T-cell response	ALL, CLL, breast, glioblastoma, lung, hepatocellular, melanoma, ovarian, sarcoma	(55, 62, 72–76)
	Cytokine production impairment		
	T-cell ”anergy” and autophagy		

ALL, acute lymphoblastic leukemia; AML, acute myeloid leukemia; CLL, chronic lymphoblastic leukemia; CML, chronic myeloid leukemia; CTLs, cytotoxic T lymphocytes; HN, head and neck; TAM, tumor-associated macrophages; APCs, antigen-presenting cells.

## Immune Evasion Mechanisms in ALL

Several studies have shown that solid and liquid tumors share immune evasion mechanisms. Studies on B cell precursor (pre-B) ALL mouse models to analyze the cytotoxicity effect of CTLs on non-immunogenic leukemic cells revealed that leukemic blasts, which are not eliminated by the initial immune response, remain in a dormant state during immune surveillance until an immune-evasive clone emerges, which requires a loss of immunogenic antigens for immune escape (77). In addition, it was proposed that ALL displayed immunological ignorance or immune tolerance (described as poor immunogenic clones that fail to alert the immune sensing mechanisms and avoid immune response) because leukemia cells lack or only a subset of them express relevant co-stimulatory accessory molecules (CD80 and CD87, respectively), showing deficient T cell activation (78, 79). Moreover, the relatively low mutation burden in ALL in comparison to other tumors could reduce neoantigen production and induce a low immunogenic response (80–82). Nevertheless, the presence of tumor-infiltrating lymphocytes as CD8+ T cells in pediatric patients with ALL suggests a potentially robust antitumor immune response (83). In addition, by predicting mutated neoepitopes in leukemia, at least one neoepitope was found in 88% of the cases, which can be recognized by CTLs and induce an anti-tumor response (84). However, B cell leukemia fails to function as an antigen-presenting cell (APC) which, in addition to its rapid dissemination, could affect the initiation and execution of anti-leukemia immunity through non-activation of T cells, which may promote immunosuppressive TME and tumor cell survival (55, 85–87). In ALL, it has been proposed that tumor-specific T cells are never properly activated; they are instead deleted or anergized upon initial antigen presentation (80, 85). Data from pre-B cells ALL show that the T cells become anergic after interleukin-10 (IL-10) expression, which is induced by CD40 activation (80, 85). Abnormal IL-10 and CD40 expression has been found in patients with ALL (78, 79, 88–90), and polymorphisms within the *IL-10* gene promoter region (-G1082A) that influences the IL-10 plasma levels have been associated with ALL prognosis (78, 91, 92).

Immune tolerance mechanisms that protect healthy tissues are hijacked by cancer to maintain immune escape through the modulation of additional processes, such as metabolically essential amino acid (tryptophan and arginine) depletion, immunosuppressive cytokine (TGF-β and IL-10) overproduction, expansion of Tregs, MDSCs, macrophages, and expression of T cell response inhibitors and co-inhibitory ligands (*e*.*g*., PD-L1) (93). Studies focused on ALL suggest that defective antigen presentation on MHC-I molecules is involved in immune evasion (94). Alterations in the expression and functionality of HLA class I (essential for CTL cytotoxicity) or II (important for CD4+ T cell response) are commonly observed in solid tumors (95, 96). The downregulation or loss of cell surface expression of HLA-I and high resistance to NK-cell-mediated killing has been described in ALL (97–101)—for instance, the C2 epitope that is encoded by HLA-C has been found to be overrepresented in patients with ALL. Given that C2 is a high-affinity ligand of the natural killer cell inhibition receptor (KIR2DL1), it has been suggested that C2 may decrease the destruction of leukemic blasts and increase the probability of late relapse in patients with ALL (>2.5 years) by reducing the cytotoxic capacity of NK cells (99). The absence of HLA class II expression in leukemic T-cells and its regulator class II trans activator has been reported (102). Recently, HLA class II expression was associated with a better prognosis in adult T cell leukemia/lymphoma (103).

**Figure 2:**
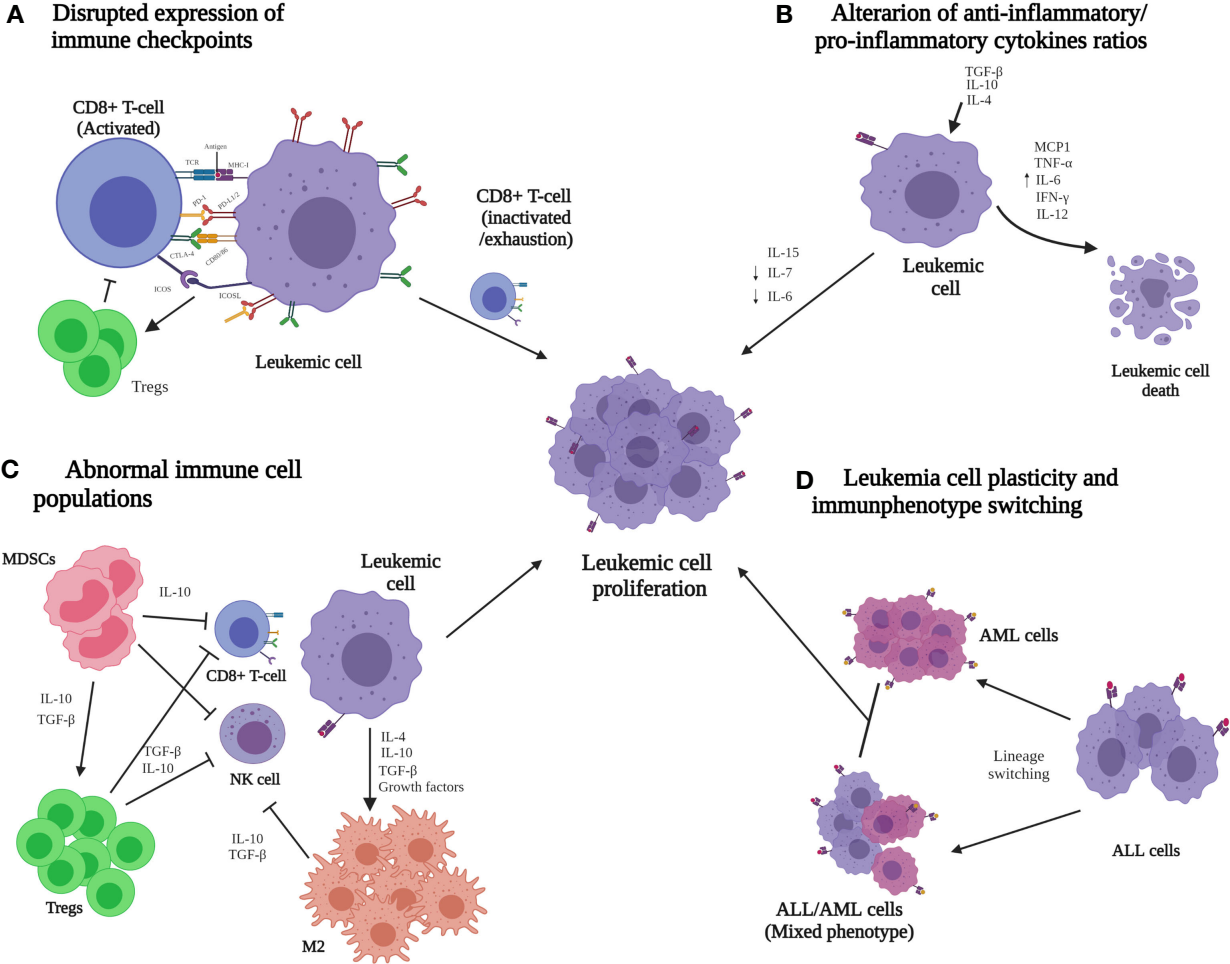
Immune evasion mechanisms that are potentially involved in the progression of acute lymphoid leukemia (ALL). **(A)** Low MHC-I and co-stimulatory ligands but high co-inhibitory lead to the inactivation or depletion of the CD8+ T-cell cytotoxic function. **(B)** Abnormal expression of anti-inflammatory cytokines (TGF-β, IL-4, and IL-10) reduces the cytotoxic T lymphocyte (CTL) population, and the pro-inflammatory cytokines (MCP1, TNF-α, IL-6, IL-12, and IFN-γ) are responsible for malignant cell destruction. **(C)** Immune cell enrichment, such as MDSCs, Tregs, and M2 macrophages, generates a favorable microenvironment for ALL cells and inhibits the activation and differentiation of CTLs and natural killer cells. **(D)** The plasticity of ALL cells leads to immunophenotype switching, which can reprogram immune evasion pathways. It has been proposed that these mechanisms could together contribute to the dissemination and progression of ALL. ALL, acute lymphoblastic leukemia; AML, acute myeloid leukemia; CD, cluster differentiation; CTLs, cytotoxic T lymphocytes; CTLA-4, cytotoxic T-lymphocyte-associated protein 4; ICOS, inducible co-stimulator; ICOS-L, inducible co-stimulator ligand; IFN-γ, interferon gamma; IL, interleukin; M2, M2 macrophages; MCP1, chemoattractant protein-1; MDSC, myeloid-derived suppressor cells; MHC-I, major histocompatibility complex class I; NK, natural killer; PD1, programmed death 1 ligand; PD-L1/2, programmed death 1/2 ligand; TCR, T-cell receptor; TNF-α, necrosis factor alpha; Treg, regulatory T-cell.

Other mechanisms, such as disrupted immune checkpoint expression and high production of suppressor factors by CTLs, alterations in the anti-inflammatory/pro-inflammatory cytokine ratio, cytotoxic abnormalities, and other cell populations with altered functions, and aberrations in the immunophenotype of the lymphoid lineage have been proposed to avoid immune surveillance by ALL cells (**Figure 2**) (85, 104).

### Disrupted Immune Checkpoint Expression and High Production of Tumor Suppressor Factors by Cytotoxic T Cells

Cytotoxic cells are the major components of the immune system that counterattack tumor cells. The overexpression of co-inhibitory ligands for specific receptors on cancer cell surfaces to disrupt T cell response is one of the primary mechanisms developed to hijack the immune system. The cell surface molecules CD28, cytotoxic T-lymphocyte-associated protein 4 (CTLA-4), inducible co-stimulator (ICOS), PD1, and PD-1L are basic ligands that induce co-stimulatory or inhibitory signals in T cells to maintain immune system homeostasis (**Figure 2A**) (105). In the early immune response stages, CD28 facilitates and maintains the CD4+ and CD8+ T cell response. CTLA-4 arrests T cell activation by triggering an inhibitory signal within the T cell, affecting critical peripheral T cell tolerance and function (106). CD28 and CTLA-4 share ligands and are necessary to avoid inappropriate or prolonged CD4+ and CD8+ T cell activation. Both molecules have been found to be constitutively expressed in acute myeloid leukemia (AML) blasts at diagnosis and have an increased expression in leukocytes from the peripheral blood of these patients compared with that of healthy controls, likely favoring AML cell escape from T cell activation and its effector functions (107). An evaluation of the CTLA-4 expression revealed that this co-inhibitory molecule was elevated in T cells in patients with high-risk ALL (108); in addition, CTLA-4 solubility was significantly elevated in 70% of B-ALL pediatric patients with active disease (109). Furthermore, CTLA-4 overexpression has been correlated with the percentage of leukemic B cells and poor prognosis in pediatric patients (108, 110), and a high serum CTLA-4 level has been detected in patients with B-ALL who died from the disease (111). Thus, the disputed CTLA-4 expression from ALL cells could be a potential mechanism of immune surveillance escape (109, 112).

PD-1 and PD-L1 overexpression has been reported to evade the host immune system in numerous cancer types (105, 110, 113). PD-L1 and PD-L2 are ligands of PD-1, which is an important inhibitory immune checkpoint that suppresses T cell activity after antigen activation. In fact, CTL function inhibition by PD-1 expression has been observed in patients with AML (113). CTLA-4 and PD-1 expression in hematological malignant cells has been suggested as an immune evasion strategy to promote leukemia blast survival and prevent efficient recognition and destruction by anti-tumor T cells (107, 110). Studies using a mouse model of disseminated AML and in transplanted patients before relapse have shown that sustained inhibitory signaling mediated by CTLA-4 and PD-1 on T cells correlates with a T cell exhaustion stage, reduced T cell effector function, and lower cytotoxicity (114, 115). Data from ALL evidenced a decrease in PD-1 expression on CD4+ and CD8+ T cells after the inhibition of myeloid–epithelial–reproductive tyrosine kinase, a gene associated with the induction of an antiapoptotic gene expression signature in B-ALL cells, leading to increased T cell activation (116). Abnormal expression of checkpoint molecule PD-1 has been reported in BM biopsies from adult patients and in T cells of pediatric cases with ALL (108, 117). In addition to the observations in ALL, upregulation of both malignant and infiltrating immune cells from B cell lymphomas and T cells of peripheral blood mononuclear cells from patients with chronic myeloid leukemia (CML) exhibits the relevance of PD-L1 in hematological malignancies (118–120). PD-L1 overexpression is correlated with poor prognosis in ALL (113) and has been found to be one of the most expressed inhibitory markers in pediatric ALL blast, whose expression is increased in relapsed patients with ALL (108, 115). Other checkpoint molecules are T cell immunoglobulin and mucin domain-containing protein 3 (TIM-3) and lymphocyte-activation gene 3 (LAG-3). TIM-3 is involved in apoptosis, and Tregs expressing TIMP3 have a higher suppressor function than Tregs negative to TIMP3 (121). LAG-3 expression has been detected in highly immunosuppressive T cells and correlates with an increased expression of IL-10 production by Tregs (122, 123). TIM-3 and LAG-3 have been found to be subexpressed in the BM of patients with ALL, in contrast to healthy subjects (117).

ICOS (CD275) is involved in maintaining immune reactions and has a relevant role in Tregs function and differentiation, which protects tumor cells from immune cells in the TME (124, 125). Significant Tregs accumulation in the BM-TME and upregulation of ICOS ligand (ICOS-L) have been observed in AML, suggesting that ICOS-L contributes to the conversion and expansion of Tregs and preserves the immunosuppressive environment. Additionally, ICOS-L and ICOS expression was found to be a predictor of OS and disease-free survival in patients with AML (126). Although there is no information regarding ICOS or ICOS-L in ALL, the relevance of ICOS in ALL immune escape is supported by studies showing that ICOS is part of the intracellular region of the signaling domain complexes that activate and induce cytotoxicity against target cells during chimeric antigen receptor (CAR) T cell immunotherapy (127).

### Alterations in the Anti-inflammatory/Pro-Inflammatory Cytokine Ratio

Inflammation is an immune response to body damage and is mediated by cytokines, which are relevant players involved in oncogenic processes, such as cell proliferation, apoptosis inhibition of mutated cells, and promotion and progression of cancer development (128). Cytokines are classified as pro-inflammatory (IL-1β, IL-6, IL-15, IL-17, IL-23, IFN-α, and TNF-α) and anti-inflammatory (IL-4, IL-10, IL-13, and TGF-β). Interestingly, an inflammatory marker analysis of neonatal blood reported that children who developed pre-B ALL had a cytokine signature (lower concentrations of the cytokine IL-8, soluble IL-6 receptor α, and TGF-β1 and higher concentrations of IL-6, IL-17, and IL-18) (129). Increased CCL2 and IL-8 concentrations of T cell-polarizing cytokines (IFN-γ and IL-12) and cytokines associated with infectious processes, such as TNF-α and IL-6, have been detected in patients with ALL at diagnosis, suggesting a pro-inflammatory state (130–133). These findings could be associated with immune cell activation by endogenous molecules that are released after tissue injury or cell death to generate an immune response against cancer (133, 134). The pro-inflammatory environment in the BM of patients with leukemia is facilitated by hematopoietic and stromal cells. However, studies indicate that cancer cells hamper immune activation by creating an anti-inflammatory TME by overproducing anti-inflammatory cytokines and by blocking the release of pro-inflammatory cytokines, thus successfully evading immune surveillance (128, 135). In CML, AML, and ALL, cancer cells express TGF-β and IL-10 to reduce immunogenicity (136, 137). Studies in mouse models with B-ALL have shown that TNF-α is secreted by B-ALL cells, and this leads to increased invasiveness and significant prolongation of surviving leukemia cells (138), which is an important mediator of leukemia-induced NK cell dysfunction. Thus, it is fundamental for NK cell immune evasion in childhood B cell ALL (139). Although IL-4 has shown antitumor effects and ALL cell suppression (140), it has been suggested that IL-4 expression in leukemia cells could reduce immunological recognition by decreasing HLA-class II molecule expression (132).

IFN-γ and interleukin 6 (IL-6) are among the most important cytokines associated with immune response in cancer (141). IFN-γ gene expression is reduced in patients with ALL, suggesting that the immune system is disrupted and leukemia cells may take advantage of defective IFN-γ production to promote escape from immune surveillance (142, 143). IL-6 contributes to lymphocyte and monocyte differentiation and induces antibody secretion by B cells. The low antibody production and decreased cellular immunity derived from abnormal IL-6 expression detected in ALL cases (**Figure 2B**), in addition to the association between single-nucleotide polymorphisms in the *IL-6* gene and susceptibility to ALL (the genotype of which correlated with IL-6 serum levels), are evidence of the relevance of this cytokine in this malignant disease (141, 144, 145).

Other cytokines and chemokines, such as IL-1, IL-7, IL-8, CCL2, CXC-10, and CXCL-12, could contribute to immunotolerance (112, 146). In fact, IL-1, IL-7, and CXCL12 expression favors ALL cell surveillance in the BM-TME (146, 147). Through the induction of CCL2 by periostin, this molecule stimulates the proliferation and dissemination of ALL (146, 148).

### Abnormal Cytotoxic and Other Cell Populations and Alteration of Their Functions

The abnormal proliferation of immune cell populations is another important mechanism for preventing immune attacks in cancer. Two distinct T cell subsets are involved in the immune system against cancer. The first is CTLs that kill cancer cells, and the second is cells required for the activation and proliferation of APCs.

Tregs (CD4+ CD25+ Foxp3+) are involved in tumor development and progression by inhibiting anti-tumor immunity in the TME (93, 149). Under physiological conditions, Tregs play an essential role in self-tolerance and immune homeostasis processes by suppressing normal and pathological immune responses and by eliminating a broad range of pathogenic microorganisms and malignant cells (85, 150–152). The correlation between tumor-infiltrating Treg levels and prognosis has been described in several malignancies, including ALL, suggesting that Tregs may be involved in the immune evasion process (149, 153–156). Indeed a high number of Tregs in the BM and peripheral blood of ALL cases has been associated with poor prognosis (153, 154, 157, 158). Studies in BM-TME have shown that immunosuppressive cytokines, such as IL-10 and TGF-β, are secreted by Tregs (**Figure 2C**) (159).

One of the biological features of patients with ALL is the presence of severe cytopenia and poor reconstitution of the innate and adaptive immune system. Although normal lymphoid and myeloid cells are present in ALL BM, the early compartment of progenitor hematopoietic cells is reduced in number and activity, including NK cells, MDSCs, and macrophages (93, 130, 160).

NK cells represent 5–20% of the lymphocytes in peripheral blood and are relevant in early antitumor immune response by lysing the tumor cells due to cytokine release. Based on CD56 and CD16 expression, two principal subpopulations of NK cells were identified: cytotoxic NK cells or cNK (CD56dim CD16+) and regulatory NK cells or NKregs (CD56highCd16-). cNK are abundant in peripheral blood (95% of the NK) and inflammation sites and show a higher cytotoxic capacity than NKregs, which predominate in lymphoid nodes (33). Natural cytotoxic receptor expression is a relevant mechanism to stimulate responses against tumor cells and has been observed to be downregulated in ALL BM (33). In recent years, several studies have provided evidence of the fundamental role of NK cells in the onset, development, and establishment of ALL (59, 139, 161, 162). ALL NK cells at diagnosis had an inhibitory phenotype associated with impaired function due to abnormal NK ligand expression (139). Torrelli et al. (59) observed a higher expression of the ligands for NK cell-activating receptors, Nec2, ULBP1, and UBLP3, on the surface of the blasts from children in contrast to adults with ALL, which could be associated with the worse clinical evolution of ALL in adults than in children (59). Differences in NK cell activity among molecular ALL subtypes have been described, with increased NK cell-activating ligand expression (NKG2D and DNAM1) in patients with ALL carrying the fusion gene *BCR-ABL* (Philadelphia chromosome: Ph+), in contrast to Ph negatives (59). Additionally, Ph+ cells are more susceptible to NK cell killing activity than ALL cells carrying no known molecular markers and were enhanced in Ph+ adult cases; B-ALL with *MLL-AF4* gene and T-ALL cases displayed a high density of the NKG2D ligand and UL16-binding protein (ULBP-1) (59). NK cells from child and adult patients have shown aberrant functions, such as low degranulation of granzyme B (117, 139). In a cohort of child patients with B cell ALL sampled at diagnosis, end induction, and maintenance, evidence of altered NK phenotype and function compared to age-matched controls was revealed. It should be emphasized that the NK abnormalities were partially corrected during the maintenance phase of the ALL treatment and were inducible in healthy NK cells after co-culture with ALL blasts *in vitro* by TGF-β1 release (139). In fact, leukemic cells secrete IL-10 and TGF-β in order to evade the effect of CTLs and NK cells (137). Furthermore, a direct contribution of the TME to the exhaustion of NK cell functions by the CRTAM/Necl-2 interaction was reported in ALL. Indeed the decreased NK cell content and their depleted cytotoxic capacity in peripheral blood are two of the predominant immune surveillance problems in acute leukemia (139). Current investigations focusing on *in vitro* activation and NK cell expansion protocols to treat ALL are underway.

MDSCs are a heterogeneous population of regulatory immature cells derived from monocytes or granulocytes that are involved in immunosuppression in patients with cancer (163). MDSCs consist of two main subpopulations (monocytic MDSCs—MO-MDSCs and polymorphonuclear MDSCs—PMN-MDSCs) that suppress the activation, proliferation, and cytotoxicity of effector T and NK cells and induce the differentiation and expansion of Tregs (163–165). The role of MDSCs in ALL remains incomplete. Recently, it was reported that patients with B cell ALL at diagnosis have a higher number of MDSCs than healthy subjects, which was even higher during induction chemotherapy (166). Furthermore, Zahran et al. (163) detected increased MDSCs in pediatric patients with B cell ALL compared to healthy controls. Moreover, these authors observed a relationship between PMN-MDSCs and the levels of peripheral and BM blast cells and CD34 + cells, suggesting that PMN-MDSC cells provide a suitable immune-suppressive state for B cell ALL tumor progression. A reduction in PMN-MDSC population was related to complete post-induction remission (163). The high number of these cells in pediatric patients with B cell ALL suggests that the increased levels and activity of MDSCs and Tregs could explain the immunosuppression state observed in this malignancy (163). MDSCs secrete TGF-β and IL-10 that have direct immunosuppressive effects and induce Treg expansion, which suppressed tumor-specific T cell responses (167) (**Figure 2C**).

Macrophages are other essential immune cell populations of the host and are composed of two subtypes: M1, which has antitumor effects, and M2 (anti-inflammatory cells with protumoral properties), which supports TME through the induction of angiogenesis, metastasis, and immune suppression (168–170). Macrophages have anti-tumoral activities at the initial stages of solid and hematological tumor development; however, TME impairs macrophage function, transforming them into immunosuppressive cell types with pro-tumoral activities (171). The frequency of M1 macrophages has been reported to be notably reduced in adult patients with B-ALL compared to controls, while M2 macrophages are increased (117). M2 is divided into several subtypes, where TAMs, which are relevant in solid tumor cell invasion, are included. Knowledge about the role of macrophages in hematopoietic malignancies has been obtained mainly from the study of lymphomas, where an association between the number of TAMs in lymph node biopsy and the prognosis of patients with classical Hodgkin lymphoma (cHL) was found (172). In ALL, the production of M2 macrophages with immunosuppressive/tolerogenic properties can be induced by tumor-mediated mechanisms (tumor-derived cytokines and growth factors, *etc.*) (171). Furthermore, it has been found that spleen leukemia-associated macrophages (LAMs) stimulate the proliferation of T cell ALL and have high migration, and their functional and phenotypic characteristics are modified by an organ-specific microenvironment (169, 173). Most LAMs have an M2-like phenotype. It has been reported that this type of immune cells also secrete immunosuppressive cytokines such as IL-10 and TGF-β (159).

### Leukemia Cell Plasticity and Immunophenotype Switching

During malignant hematopoietic disorders, such as acute leukemias, intrinsic and extrinsic signals (including those participating in immune surveillance) influence the cell differentiation pathway and cooperate in abnormal fate decisions, highlighting the relevance of a continuous homeostatic control to produce elements of tumor suppression (174, 175). Switching of CML to ALL in the blast crisis, AML cases relapsing as ALL, ALL converting AML after chemotherapy, and mixed phenotypes (simultaneous expression of both myeloid and lymphoid antigens) suggest that linage-associated molecule expression contributes to immune response disruption and facilitates cancer progression (174, 176–179). Lineage switching in leukemia is more frequent in children than in adults, and most cases are ALL converting to AML (180). Studies have proposed that this process is a consequence of stem cell plasticity (capacity to cell fate conversion in defined cells adopting biological properties to the same or different lineages) since the evidence shows that cancer cells are derived from the same founder clone in leukemia lineage switching (180). Leukemias with lineage switching appear to be more common in specific genetic subtypes, such as those with *KMT2A* (*MLL*) gene rearrangements (180). The absence of *EBF1* expression in ALL allows early lymphoid progenitors to differentiate into the myeloid lineage, and deletion of *PAX5* in mature B cells can induce conversion to different fates, including macrophages and T cells (176, 181). Low *PAX5* expression has been reported in patients with ALL and very early relapse-expressing AML genes, such as *MPO* and *FLT3* (174). A single-cell RNA-seq study revealed that plasticity coexists with oncogenic and immune evasion programs in early T progenitor ALL (174), suggesting that specific features acquired during lineage conversion could contribute to immune evasion response in ALL. It has been proposed that the plasticity of leukemic blasts in early progenitor T cell ALL can modulate the treatment based on inhibitors of the Notch pathway due to the coexistence of transcriptional programs that are characteristic of lymphoid and myeloid lineages. Additionally, immunoevasion signatures were found to be activated in the TME—for example, the interaction between hepatitis A cellular virus receptor 2 and galectin 9 is associated with CD8+ T cell dysfunction (174). Studies aimed at understanding leukemic blast plasticity could contribute to the identification of potential therapeutic targets based on the reversion of T cell depletion and consequently improve OS rates in patients with ALL.

## Bone Marrow Tumor Microenvironment and Immune System Evasion

Immunological evasion is due to mechanisms inherent to the TME. It is well known that malignant blasts maintain a close interaction with normal cells within the BM niche and, at the expense of normal hematopoiesis, remodel functionally and structurally the BM-TME to favor ALL development and promote tumor cell dissemination and chemotherapy resistance (43, 47, 55, 112, 118, 182–184). BM-TME favors tumor growth through polarization of host immunity to prevent anti-cancer immune responses. Alterations in immune cell populations in the TME are other mechanisms involved in the immune evasion by leukemic cells—for instance, it has been reported that the presence of leukemic cells in BM affects the CD14-expressing monocytes and non-classical CD16-expressing monocytes populations (185). Leukemic blasts have the capacity for TME remodeling during disease progression and promote monocyte differentiation into non-classical monocytes. In the BM-TME, a decrease in CTLs and NK cells has also been reported as well as an increase in suppressor immune cell populations such as Tregs, M2 macrophages, and MDSCs to support an immunosuppressive microenvironment (55). Mice models of AML revealed that those leukemic cells reduce the osteoblast population, modifying the lineage fate of hematopoietic stem cells, which increase tumor burden and reduce OS (186, 187). The interaction between leukemic blasts and the different cell types has been associated with a major surveillance of tumor cells (188). Besides this, ALL cells and the primary mesenchymal stromal cells (MSC) within the niche interact by using tunneling nanotubes (TN) that induce the secretion of prosurvival cytokines IL-8, CCL2, and CXCL10, driving stroma-mediated steroid resistance. By interruption of the TN signal, the leukemogenic processes are inhibited; thus, pre-B-leukemic cells are resensitized with prednisolone (189, 190).

ALL blasts also express surface molecules shared with hematopoietic stem cells and interact with extracellular matrix (ECM) molecules, soluble factors, and cytokines for ALL promotion—for instance, it has been reported that integrins have a role in the retention of leukemic blasts in the BM and contribute to ALL dissemination from BM to the CNS and chemoresistance (191, 192). Otherwise, the MSC-derived ECM proteins, such as periostin and osteopontin in the niche, stimulate the proliferation and dissemination of ALL. ECM of the BM also represents a physical barrier that contributes to immune evasion in the cell niche (193).

Another important alteration in the leukemic TME is the increased levels of anti-inflammatory and immunosuppressive cytokines, such as IL-10 and TGF-β, and the high expression of PD-1 and TIGIT, which contribute to tumor progression and immune evasion (43, 47, 55, 194). IL-1, IFN-γ, TNF-α, and HLA-G in the BM-TME may induce immune tolerance and then ALL recurrence. Additionally, overexpression in BM-TME of chemotactic cytokines such as CXCL12/CXCR4 and CCL25/CCR9 (produced by stromal cells in the BM) has a role in ALL and influences the outcomes and chemoresistance. Thus, targeting the chemokine axis could significantly reduce tumor burden in ALL (182).

It is well known that the immunosuppressive microenvironment surrounding tumor cells represents a key cause of treatment failure; therefore, BM-TME is the central target for reprogramming the immune system in ALL and other hematological malignancies.

## Into the Frontline of ALL Treatment: Targeting the Immune Cells

Initial ALL treatment comprises induction, consolidation, and long-term maintenance therapy. The backbone of ALL therapy is chemotherapy using drugs developed during the 1950s and 1960s focused on leukemic cell eradication, normal hematopoiesis restoration, and prevention of “sanctuary site” invasion, relapse, and death (195, 196). Chemotherapy has achieved considerable success in ALL survival (9); however, relapse occurring in approximately 20% of patients with ALL is the main obstacle in improving the OS rates. Adult patients with ALL have a higher risk of relapse than pediatric patients, and the protocols used (adapted from pediatric protocols) reach less than 50% of the success rate and have lower minimal residual disease negative rates after induction therapy (197). Allogeneic hematopoietic stem cell transplantation (alloHSCT) has been an effective anti-leukemic therapy for patients with ALL (198, 199) but is a highly toxic therapy, and disease recurrences can occur within the time of immunosuppressive treatment (200, 201).

Over the last two decades, multiple studies have attempted to improve the OS of patients with ALL by incorporating new agents into the treatment protocols and exploiting the immune response against leukemia cells. Targeting of tumor cells is a promising therapeutic approach. Antibody-based therapeutic strategies are being developed to select cells of the immune system, enhance anti-tumor immune response, and reduce damage to normal tissues (93, 202). T cell signaling pathway inhibition (particularly PD-L1/PD-1), immune cell regulation, and the prevention of tryptophan depletion by indoeamine-2,3-dioxygenase are the most well-studied immunosuppressive mechanisms in liquid tumors (93). Nevertheless, few trials based on these pathways have been described for ALL treatment. Monoclonal antibodies (mAbs), immune checkpoint blockers, CAR T cells, and bispecific T-cell engagers (BiTEs) are currently used in ALL treatments approved by the USA Food and Drug Administration (FDA) (203–205). Approaches using adoptive T cell therapy (ACT) and tumor neoantigens are under investigation (93).

### Monoclonal Antibodies

Antibodies are the basis for many new anti-cancer treatment strategies due to their immunomodulatory properties and capacity to promote the induction of anti-tumor immune responses. These antibodies target self-tumor antigens or the TME to inhibit tumor growth by increasing host immune responses to antigens expressed by the tumor itself or by reducing pro-tumorigenic factors generated in the tumor stroma (206). CTLA-4-specific mAbs have been used in human cancers, such as melanoma (206). Using the anti-CTLA-4 mAb (ipilimumab) in combination with IgG4 mAb (nivolumab), which disrupts the interaction between PD-1 and PD-L1/PD-L2, in patients with relapsed/refractory cHL, non-Hodgkin lymphoma, or multiple myeloma showed no significant improvement in efficacy over single-agent nivolumab (207). The anti-PD-1 anti-leukemic treatment is based on the maintenance and expansion of tumor-specific memory T cells and NK cell activation. This approach has been explored in diverse tumors, including relapsed/refractory lymphoid malignancies; however, its clinical application in ALL remains unknown. In ALL, CD-38 and CD-52 have been identified as target antigens of mAbs for the treatment of relapsed T cell ALL. Currently, there are ongoing clinical trials testing the efficacy of anti-CD38 mAbs (isatuximab and daratumumab) and anti-CD52 mAbs (alemtuzumab) with favorable results for better disease prognosis (127).

### Bispecific T Cell Engagers

BiTEs are bispecific recombinant glycoproteins with two single-chain variable fragments (scFvs) connected by a flexible linker, whose targets are membrane molecules (costimulators, coinhibitors, adhesion, *etc.*) from both T cells and malignant cells. BiTEs favor immune responses by creating an immune synapse among tumor antigens and T cells (204). The distribution of BiTEs depends on factors such as the diffusion of the vascular endothelium, laminar flux rate, and interaction with the target. Blinatumomab (anti-CD19/anti-CD3; AMG103) is a BiTE that binds to CD3+ lymphocyte T and CD19+ B lymphocytes. Although blinatumomab induces selective lysis of tumoral cells, its half-life is short, and constant administration is necessary for effect maintenance. The Children’s Oncology Group has incorporated blinatumomab in clinical trials in patients with B cell ALL with a standard risk (1–9.99 years and leukocyte count <50,000/ml) classification. Good results and acceptable toxicity were observed; in addition, half of the population had a significant twofold improvement in median OS compared to patients with standard chemotherapy regimens (208).

Studies in patients with relapse/refractory pre-B cell ALL indicate that Treg proportion could determine the prognosis of blinatumomab treatment. It was observed that blinatumomab responders had a lower percentage of Tregs (4.82%) in peripheral blood compared to non-responders (10.25%). Additionally, the restoration of the activated T cell population was detected after the *in vitro* depletion of Tregs in leukemic blasts, thus highlighting the regulatory role of Tregs in the development of the immune response in ALL (209).

The main disadvantage of BiTEs is the induction of cytokine release syndrome (CRS) through proinflammatory cytokines and aplasia of lymphocytes B (205). Although collateral effects in clinical assays with blinatumomab have been reported (fever, nausea, headache, neurological, and hepatic adverse effects), a lower percentage of minimal residual disease in patients treated with blinatumomab in contrast to patients with high-risk ALL treated with conventional regimens was reported (204, 208, 210).

### CAR T Cells

CAR is a synthetic construct formed by an extracellular scFvc (that recognizes the tumor antigen) fused to a transmembrane domain and to intracellular activating/co-stimulator motives (CD3ζ, CD28, 4-1BB) (204, 211–213). CAR is transduced to T cells (CAR T cell), and after the recognition of the tumoral antigen, it promotes a cytotoxic effect on the target cell (205). It has been reported that CAR T cells act independently of HLA recognition. Therefore, this approach could be used in different cases to overcome the lower HLA density of ALL malignant cells. Furthermore, it is feasible to use CD4+ T and CD8+ T cells to generate CAR T cells, which increases the effector and cytotoxic potential of T cells (213).

CAR T cells have recently been approved by the FDA to treat patients with leukemia and lymphoma (203–205), and several clinical trials of CD19 CAR T cell therapy are being carried out in ALL relapse patients, which have shown favorable results with remission after 6 months in up to 90% of the patients, with 78% OS and 67% event-free survival (214). However, in a group of refractory/relapsed patients with B cell ALL, CD22 CAR T cell therapy treatment achieved a complete remission of 70.5% in comparison with those previously treated with CD19 CAR T cells without success. CD22 CAR T cell-treated patients only exhibited a moderate grade of CRS and neurotoxicity, and better results were observed in patients undergoing alloHSCT (215). An important advantage of CAR T cells is their capacity to interact with malignant cells displaying different antigens, such as CD19, CD20, and CD22 (212).

Although CAR T cell implementation in B cell ALL has obtained favorable results, its implementation in T cell ALL presents limitations because CAR T cells and malignant T cells share similar expression profiles of target antigens, which gives rise to a non-specific cytotoxic activity incapable of discriminating CAR T cells from malignant T cells, leading to T cell aplasia and eventual immunodeficiency. Notwithstanding, the identification of antigens, such as CD4, CD5, and CD7, on T cell ALL shows promise for the use of CAR T cell technology, the clinical trials of which are ongoing (127).

Limitations of CAR T cell therapy in ALL involve the following (1): poor expansion and limited persistence *in vivo* caused by defects in the design and manufacturing of CAR T cell therapy (2), internalization of the CD19 glycoprotein and resurgence in tumor cells (3), toxicity (CAR T cells present numerous cellular interactions that could promote the cytokine-mediated systemic inflammatory response), and (4) aplasia of B cells and humoral deficiency that might promote infections (204, 205, 216).

### Adoptive T Cell Therapy Using Tumor Neoantigens

ACT is based on TIL expansion and infusion in patients following lymphodepletion. ACT aims to generate a robust immune-mediated antitumor response *via* infusion of *ex vivo*-manipulated T cells. Studies have suggested that clinical outcomes correlate with tumor mutational and neoantigen load (217–219). Although ALL has been described as a malignancy with low mutational load, a recent analysis reported that it is possible to obtain immunodominant neoantigens that could be used to develop neoepitope-CD8+ T cells and treat patients with ALL (83, 220). To explore the effectiveness of this strategy, 36 putative neoantigens from the *ETV6*–*RUNX1* fusion were tested, and 31 neoantigens were immunogenic. The co-culture of HLA-specific APCs with neoepitopes and isolated CD8+ tumor-infiltrating lymphocytes results in TNF-α and IFN-γ production. Therefore, this strategy provides a possibility to consider the adoptive transfer of neoepitope-CD8+ T cells as immunotherapy in leukemia and could be used in the consolidation phase or subsequent treatment (83).

### Activation of Necroptosis

The suppression of cell proliferation in leukemic lineages has significant challenges. On one hand, mAb treatment ablates the main elements of the adaptive immune response, including T and B cells, which could favor infection burst or immune-mediated disease development (221, 222). On the other hand, to increase tumor-specific T cell responses, it is necessary to promote leukemic cell immunogenicity. To date, the primary goal of several research groups has been to promote apoptosis in malignant cells; however, this type of cell death is immune-tolerogenic. Recent studies have shown that a new class of targeted drugs (second mitochondrial activator of caspases, SMAC) antagonizes diverse anti-apoptotic proteins (inhibitor of apoptosis proteins) and, in combination with dexamethasone, promotes increased immunogenic cell death (necroptosis) in ALL (223, 224). Necroptosis is a regulated inflammatory mode of cell death that is caspase-independent and presents highly regulated necrotic features (225). Necroptosis produces the release of DAMPs and proinflammatory cytokines, allowing a better cytotoxic function by tumor-specific T cells (225–227). The activation of necroptosis has been explored as an anti-leukemic therapy, and several SMAC mimetic compounds are currently in phase I or II clinical trials to treat hematological malignancies, including ALL (225, 228). The leading problem with active necroptosis in ALL therapy is its potential to induce immunogenicity. Observations from solid tumors suggest that necroptosis is not always pro-inflammatory or immunogenic; however, there are no reports of necroptosis in ALL (223, 229).

## Conclusion

Despite defined treatment protocols, leukemia remains a global health problem due to high relapse and treatment failure rates. ALL studies suggest that immune response evasion by leukemic cells could promote malignant cell proliferation and invasion. The identification of leukemic cell strategies to deactivate immune cells and induce an immunosuppressive TME to resist apoptosis has been suggested to have potential implications in the field of personalized immunotherapy for ALL—for example, the infusion of co-stimulatory adapted CAR T cells to increase cytotoxic T cell responses is a current option for ALL treatment. The infusion of neoepitope-specific ALL cells to increase the MHC response is also a potential alternative. Among the treatments for patients with ALL, the induction of leukemic cells to become immunogenic is a promising alternative because it promotes an immunogenic microenvironment and influences direct malignant cell elimination. Further research into the immune evasion mechanisms underlying ALL development and progression is required to gain knowledge on the molecular and cellular leukemogenesis mechanisms, which could contribute to the design of new anti-ALL therapies.

## Author Contributions

SJ-M, IA-U, and CP-A participated in the preparation of the original draft. JR-B and AH-M participated in revision and editing. SJ-M took charge of conceptualization, supervision, and funding acquisition. All authors contributed to the article and approved the submitted version.

## Funding

This work was supported by the Consejo Nacional de Ciencia y Tecnología (CONACyT, Investigación en Fronteras de la Ciencia—2016-01-2119) and by the National Institute of Genomic Medicine (01/2018/I and 19/2019/I). ISA-U and CJP-A were supported by CONACyT with the scholarships CVU 324181 and 821714, respectively.

## Conflict of Interest

The authors declare that the research was conducted in the absence of any commercial or financial relationships that could be construed as a potential conflict of interest.

## Publisher’s Note

All claims expressed in this article are solely those of the authors and do not necessarily represent those of their affiliated organizations, or those of the publisher, the editors and the reviewers. Any product that may be evaluated in this article, or claim that may be made by its manufacturer, is not guaranteed or endorsed by the publisher.

## Glossary

**Table:**

ACT	adoptive T cell therapy
ALL	acute lymphoblastic leukemia
alloHSCT	allogeneic hematopoietic stem cell transplantation
AML	acute myeloid leukemia
APCs	antigen-presenting cells
BiTEs	bispecific T-cell engagers
CAR	chimeric antigen receptor
cHL	classical Hodgkin lymphoma
CML	chronic myeloid leukemia
CTLA-4	cytotoxic T-lymphocyte-associated protein 4
CTLs	cytotoxic T cells
CXCL	IFNγ-inducible chemokine (C-X-C motif) ligands
DAMPs	damage-associated molecular patterns
EBF1	EBF transcription factor 1
ETV6	ETS variant transcription factor 6
FDA	Food and Drug Administration
FLT3	Fms-related receptor tyrosine kinase 3
HLA	human leukocyte antigen
HLA-G	human leukocyte antigen G
IAP: ICOS	inducible co-stimulator
ICOS-L	ICOS ligand
IFN-γ	interferon gamma
IL	interleukin
INF-α	interferon alpha
KIR2DL1	natural killer cell inhibition receptor
KMT2A	lysine methyltransferase 2A
LAG-3	lymphocyte-activation gene 3
LAMs	leukemia-associated macrophages
mAbs	monoclonal anti-bodies
MSCs	mesenchymal stromal cells
MDSCs	myeloid-derived suppressor cells
MHC	major histocompatibility complex
MLL	lysine methyltransferase 2A
MPO	myeloperoxidase
NHL	non-Hodgkin lymphoma
NK	natural killer
NKG2D	natural killer group 2 member D
NKT	natural killer T
OS	overall survival
PAX5	paired box 5
PD1	programmed death 1
PD-L1	programmed death 1 ligand
pre-B	B cell precursor
RUNX1	RUNX family transcription factor 1
SAP	(SLAM)-associated protein
scFvs	single-chain variable fragment
SMAC	second mitochondrial activator of caspases
TAMs	tumor-associated macrophages
TIM-3	T-cell immunoglobulin and mucin domain-containing protein 3
Tregs	regulatory T cells
TILs	tumor-infiltrating lymphocytes
TME	tumor microenvironment
TNF	tumor necrosis factor
TRAIL	TNF-related apoptosis-inducing ligand
TN	tunneling nanotubes
ULBP-1	UL16-binding protein
